# Dexmedetomidine as Adjunctive Therapy for the Treatment of Alcohol Withdrawal Syndrome: A Systematic Review and Meta-Analysis

**DOI:** 10.3390/ph17091125

**Published:** 2024-08-26

**Authors:** Marco Fiore, Aniello Alfieri, Giacomo Torretta, Maria Beatrice Passavanti, Pasquale Sansone, Vincenzo Pota, Vittorio Simeon, Paolo Chiodini, Antonio Corrente, Maria Caterina Pace

**Affiliations:** 1 Department of Women, Child and General and Specialized Surgery, University of Campania “Luigi Vanvitelli”, Via Luigi De Crecchio, 2, 80138 Naples, Italy; anielloalfieri@gmail.com (A.A.); beatrice.passavanti@libero.it (M.B.P.); pasquale.sansone@unicampania.it (P.S.); vincenzo.pota@unicampania.it (V.P.); antonio.corrente.md@gmail.com (A.C.); caterina.pace@libero.it (M.C.P.); 2 Department of Anesthesiology and Reanimation, “San Giuseppe Moscati” Hospital, 83100 Avellino, Italy; giacomotorretta1989@gmail.com; 3 Department of Mental, Physical Health and Preventive Medicine, University of Campania “Luigi Vanvitelli”, Largo Madonna Delle Grazie, 1, 80138 Naples, Italy; vittorio.simeon@unicampania.it (V.S.); paolo.chiodini@unicampania.it (P.C.)

**Keywords:** dexmedetomidine, alcohol withdrawal syndrome, intensive care, sedatives, systematic review

## Abstract

Alcohol withdrawal syndrome (AWS) is defined as the cessation or reduction in heavy and prolonged alcohol use within several hours to a few days of cessation. The recommended first-line therapy for AWS ranging from mild to severe or complicated remains benzodiazepines; in cases where benzodiazepines are not adequate in controlling persistent autonomic hyperactivity or anxiety, dexmedetomidine could be utilized. The possible advantage of dexmedetomidine compared to benzodiazepines is that it does not cause respiratory depression, thus reducing the risk of intubation and hospitalization in the ICUs, with the potential reduction in healthcare costs. The purpose of this systematic review and meta-analysis (PROSPERO CRD42018084370) is to evaluate the effectiveness and safety of dexmedetomidine as adjunctive therapy to the standard of care for the treatment of AWS. We retrieved literature from PubMed, EMBASE, and CENTRAL until 10 January 2024. Eligible studies were both randomized trials and nonrandomised studies with a control group, published in the English language and peer-reviewed journals. The primary outcome was tracheal intubation; secondary outcomes were (i) bradycardia and (ii) hypotension. A total of 3585 papers were retrieved: 2635 from EMBASE, 930 from Medline, and 20 from CENTRAL. After eliminating duplicates, 2960 papers were screened by title and abstract; 75 out of the 2960 papers were read in full text. The qualitative synthesis included nine of all manuscripts read in full text. The quantitative synthesis included eight studies for the primary outcome (tracheal intubation), seven for the secondary outcome bradycardia, and six for the secondary outcome hypotension. The meta-analysis showed that Dexmedetomidine, as adjunctive therapy, is not more effective than standard therapy in reducing the risk of tracheal intubation in AWS [RR: 0.57, 95% CI: 0.25–1.3, *p* = 0.15]. It also appears to be less safe than sedative therapy as it significantly increases the risk of bradycardia [RR: 2.68, 95% CI: 1.79–4.16, *p* = 0.0016]. Hypotension was not significantly different in patients who received dexmedetomidine [RR: 1.5, 95% CI: 0.69–3.49, *p* = 0.21].

## 1. Introduction

Alcohol withdrawal syndrome (AWS) is defined as a set of symptoms that occur after the cessation or reduction in heavy and prolonged alcohol use within several hours to a few days of cessation. It will start manifesting with two or more of the following symptoms: autonomic hyperactivity, increased hand tremors, insomnia, nausea/vomiting, transient visual/auditory/tactile hallucinations or illusions, psychomotor agitation, anxiety, or generalized tonic–clonic seizures [1].

The recommended first-line therapy for AWS ranging from mild to severe or complicated remains benzodiazepines; in cases where benzodiazepines are not adequate in controlling persistent autonomic hyperactivity or anxiety, Alpha-2 agonists such as dexmedetomidine could be utilized (recommendation V.36) [2]. The ability of dexmedetomidine is unique in that it can provide both sedation and analgesia without respiratory depression [3]. The distribution of dexmedetomidine is rapid, and in the liver, mainly through glucuronidation and hydroxylation, inactive metabolites are formed. The pharmacokinetics of dexmedetomidine are highly variable among individuals, especially in critically ill patients. Dexmedetomidine stops the propagation of pain signals by binding to presynaptic alpha-2 adrenoceptors and preventing norepinephrine from being released.

Furthermore, the anti-inflammatory state seems to be favorably influenced by dexmedetomidine, which reduces the levels of inflammatory cytokines. However, further research should be conducted on the association between dexmedetomidine and these outcomes [4].

Hospitalizations for substance use disorders are often associated with alcohol, which is the most common substance implicated; it is the most prevalent drug in emergency department (ED) visits, followed by opioids (14.07%), methamphetamine (11.02%), marijuana (10.78%), and cocaine (4.71%) [5]. The estimated medical costs for alcohol-related disorders in U.S. hospitals are $7.6 billion annually [6]. More frequent use of intensive care units (ICUs) by institutions leads to increased invasive procedures and higher hospital costs, without improving in-hospital mortality [7].

The possible advantage of dexmedetomidine compared to benzodiazepines is that it does not cause respiratory depression, thus reducing the risk of intubation and hospitalization in the ICUs, with the potential reduction in healthcare costs.

## 2. Results

**Figure 1:**
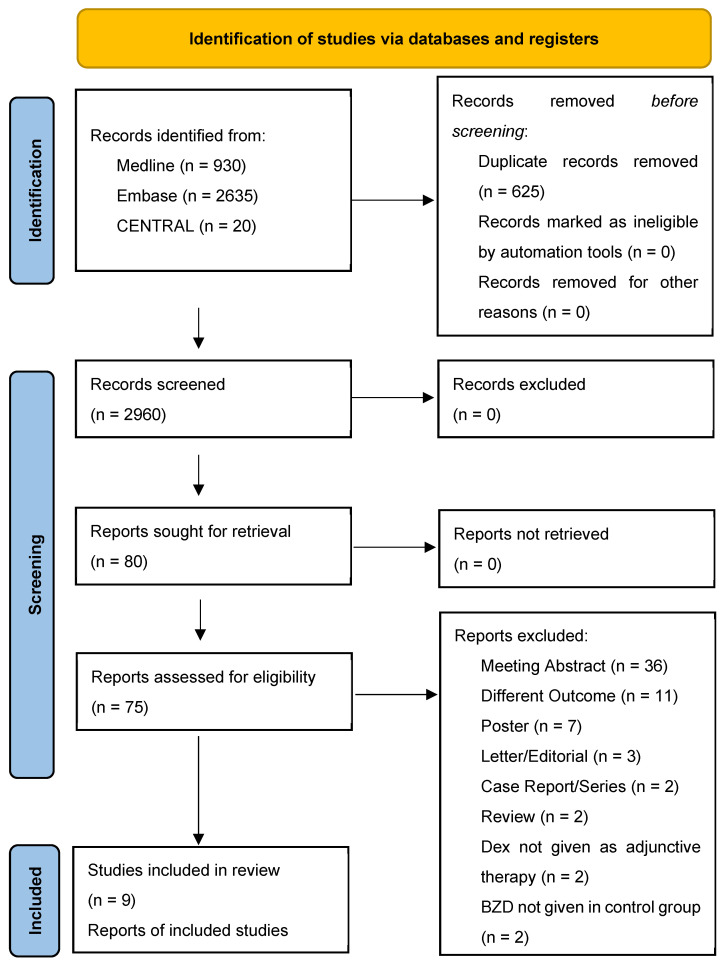
PRISMA 2020 flow diagram for new systematic reviews, which included searches of databases and registers only.

A total of 3585 papers were retrieved: 2635 from EMBASE, 930 from Medline (via Ovid), and 20 from CENTRAL. After eliminating duplicates, 2960 papers were screened by title and abstract; 75 out of the 2960 papers were read in full text (Figure 1). The qualitative synthesis has included nine out of all manuscripts read in full text (Table 1). Of the nine studies included in the qualitative synthesis, two were RCT, and seven were retrospective cohort studies. The overall risk of bias of the two RCT was low (Table 2); the quality of the seven retrospective studies, assessed using the Newcastle-Ottawa Scale [8], was moderate-high (Table 3).

### 2.1. Primary Outcome (Tracheal Intubation)

**Figure 2:**
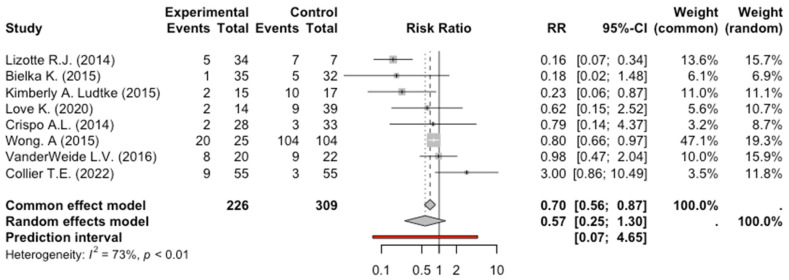
Forest Plot Intubation [9,10,11,12,13,14,16,17].

The quantitative synthesis included eight studies for the primary outcome (tracheal intubation), seven for the secondary outcome bradycardia, and six for the secondary outcome hypotension. The total number of patients enrolled in the eight studies exploring tracheal intubation was 535: 226 patients received dexmedetomidine and 309 received benzodiazepines or benzodiazepines plus propofol. Of these 226 patients who received dexmedetomidine, 49 were intubated, while 150 patients in the control group were intubated. The meta-analysis of the eight studies evaluating tracheal intubation did not show a significant intubation reduction in AWS patients receiving dexmedetomidine, RR: 0.57, 95% CI: 0.25–1.3, *p* = 0.15. The degree of heterogeneity across studies was high (I^2^ = 73%, *p* < 0.01) (Figure 2).

### 2.2. Secondary Outcome (Bradycardia)

**Figure 3:**
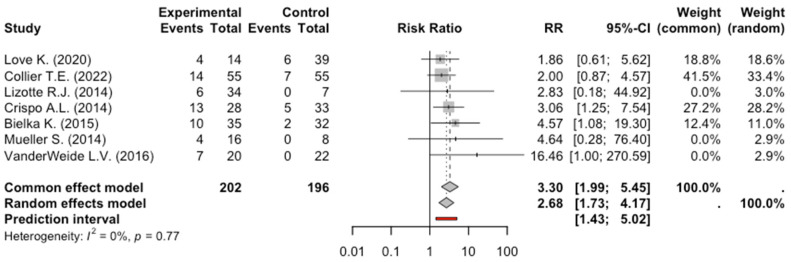
Forest Plot Bradycardia [9,10,11,12,13,14].

The total number of patients enrolled in the 7 studies exploring Bradycardia was 398: 202 patients received dexmedetomidine, and 196 received benzodiazepines or benzodiazepines plus propofol. Of these 202 patients who received dexmedetomidine, 58 presented with bradycardia, while 20 patients were in the control group. The meta-analysis of the studies evaluating bradycardia showed a significant risk in AWS patients receiving dexmedetomidine, RR: 2.68, 95% CI: 1.79–4.16, *p* = 0.0016. The degree of heterogeneity across studies was low (I^2^ = 0%, *p* = 0.77) (Figure 3).

### 2.3. Secondary Outcome (Hypotension)

**Figure 4:**
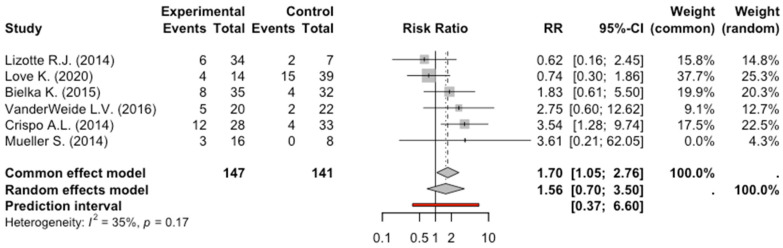
Forest Plot Hypotension [9,10,11,12,13,15].

The total number of patients enrolled in the six studies exploring hypotension was 288: 147 patients received dexmedetomidine and 141 received other sedative medications. Of these 147 patients who received dexmedetomidine, 38 had arterial hypotension, while 27 patients were in the control group. The meta-analysis of the six studies evaluating hypotension did not show a significant reduction in arterial pressure in patients who received dexmedetomidine, RR: 1.5, 95% CI: 0.69–3.49, *p* = 0.21. The degree of heterogeneity across studies was moderate (I^2^ = 35%, *p* = 0.17) (Figure 4).

## 3. Discussion

It has been shown in experimental studies and single-case reports that 2-agonist dexmedetomidine has a beneficial effect on managing the autonomic symptoms experienced with AWS [18,19,20].

It is not uncommon for patients with AWS to have to increase their doses of benzodiazepines and require tracheal intubation to protect their airways. Observational studies suggest that dexmedetomidine therapy for AWS is associated with substantially reduced benzodiazepine dosing and a decreased hyperadrenergic cardiovascular response to ethanol abstinence with a low rate of intubation and mechanical ventilation [21,22].

An unregistered systematic review and meta-analysis, with a precedent search date, found that bradycardia and hypotension incidence significantly favored the benzodiazepine arm in both subgroups [23].

In conclusion, the hypothetical benefit of adding dexmedetomidine to sedatives in the treatment of AWS was not observed in our meta-analysis; in addition to having no benefit in terms of efficacy and not reducing the risk of intubation of AWS patients, dexmedetomidine is less safe than sedatives because it significantly increases the risk of bradycardia. In most studies, there was no mention of the severity of bradycardia and related management. Lizotte et al. found that bradycardia did not resolve unless the infusion rate was decreased or the infusion was stopped [12].

## 4. Methods

The systematic review protocol was registered in PROSPERO (CRD42018084370) and fully published in the Joanna Briggs Institute database of systematic reviews and implementation reports [24].

### 4.1. Study Search

The PICO method was used to perform the search strategy (Table 4). The databases of the search included MEDLINE via PubMed, EMBASE, and the Cochrane Central Register of Controlled Trials (CENTRAL). The search was conducted from inception to 10 January 2024.

### 4.2. Study Selection

After the search, we removed the duplicate and listed all included studies using citation management software (Endnote VX9.3.3 Clarivate Analytics, Philadelphia, PA, USA). We included both randomized clinical trials (RCT) and non-randomized studies with a control group, published in peer-reviewed journals in the English language as eligible studies. No restriction on the time of publication was applied. Two authors (A.A. and G.T.) independently evaluated the eligible studies with an initial screening based on the title and abstract. The above-mentioned authors followed by a full-text screening of the selected articles for final inclusion. A third author (M.F.) resolved any disagreements on study eligibility or data extraction. The full text of the selected citations was assessed in detail by two independent reviewers (A.A. and G.T.) who recorded the reasons for the exclusion of full-text studies that did not meet the inclusion criteria of the systematic review, and a final check was conducted by a third one (M.F.). In the final report, the results of every step of the planned search are presented in their entirety and presented in a flow diagram for Preferred Reporting Items for Systematic Reviews and Meta-analyses (PRISMA) [25].

### 4.3. Definition and Outcome

For this study, we defined experimental therapy as the combined use of Dexmedetomidine and other sedatives, and control therapy as the use of benzodiazepine as a single sedative therapy or in association with other sedatives. The primary outcome was tracheal intubation. The secondary outcomes were 1. hypotension and 2. bradycardia, respectively. For the secondary outcomes, we used the definitions provided by the authors of the included studies. All the outcomes were evaluated in patients who had a diagnosis of AWS.

### 4.4. Data Extraction and Quality Assessment

Data were extracted from studies included in the review by two reviewers independently (A.A., G.T.) using the Cochrane data collection form for intervention reviews for RCTs and non-RCTs; the two authors assessed the methodological quality of the included studies (A.A., G.T.). The risk of bias of enrolled RCTs was evaluated using the Cochrane Collaboration Revised Assessment Tool (RoB 2) [10,26]. The quality of nonrandomized studies was assessed using the Newcastle–Ottawa Assessment Scale (NOS) [27].

### 4.5. Data Analysis

We performed a meta-analysis using the Mantel-Haenszel method to calculate the (fixed-effect) weights of studies with binary outcome data [28]. Furthermore, a more conservative approach (inverse variance method) with the random-effects estimates of risk ratio (RR) for each outcome, which allow for the variation of real effects across studies, was taken as “main results” [29]. We quantified heterogeneity using the I^2^ statistic, which describes the percentage of total variation across studies that was attributable to heterogeneity rather than to chance. I^2^ values of 25%, 50%, and 75% correspond to cut-off points for low, moderate, and high degrees of heterogeneity. We quantified the between-study variance with the Paule–Mandel Estimator, which assumes that the true effect sizes of the studies are normally distributed around some overall effect size. It is a method for estimating τ^2^, particularly in the context of a random-effects model in meta-analysis. It was preferred because it can provide more reliable estimates of τ^2^ in meta-analyses with a small number of studies [30]. The Q-Profile method was used to construct confidence intervals for τ^2^ and τ. The Hartung–Knapp adjustment was used to improve the accuracy of confidence intervals for the overall effect size. To deal with zero cell frequencies, a small number (0.5) was added to all cells of the contingency table, including those with zero values (Continuity Correction of 0.5). All analyses were performed using R software version 4.3.3 (29 February 2024) “Angel Food Cake” [8].

## 5. Conclusions

Dexmedetomidine, as adjunctive therapy, is not more effective than standard sedative therapy in reducing the risk of tracheal intubation in AWS patients. It also appears to be less safe than sedative therapy as it significantly increases the risk of bradycardia.

## Figures and Tables

**Table 1:** Studies included in the quantitative synthesis.

Author (Published Year) [Ref.]	Setting	Study Design	Control Group	Time Spam	N. Patients	Outcome(s)
Crispo, A.L. (2014) [9]	ED	RC	BZD	2011–2012	122	TI + B + H
Bielka, K. (2015) [10]	ED	RCT	BZD	NS	134	TI + B + H
VanderWeide, L.A. (2016) [11]	Mixed	RC	BZD	2008–2012	84	TI + B + H
Lizotte, R.J. (2014) [12]	ED	RC	BZD + P	2010–2013	82	TI + B + H
Love, K. (2020) [13]	ICU	RC	BZD + P	2015–2018	53	TI + B + H
Collier, T.E. (2022) [14]	ICU	RC	BZD	2015–2018	110	TI + B
Mueller, S. (2014) [15]	ED	RCT	BZD	2009–2012	48	B + H
Ludtke, K.A. (2015) [16]	ED	RC	BZD + P	2002–2009	64	TI
Wong, A. (2015) [17]	Mixed	RC	BZD + P	2009–2012	258	TI

B: bradycardia; BZD: benzodiazepine; ED: emergency department; H: hypotension; ICU: intensive care unit; NS: not specified; P: propofol; RC: retrospective cohort study; RCT: randomized controlled trial; TI: tracheal intubation.

**Table 2:** Risk-of-bias assessment in a systematic review of randomized trials, using version 2 of the Cochrane risk-of-bias tool.

Study	Ref.	R	D	Mi	Me	S	O
Bielka, K.	[10]	+	−	+	+	+	+
Mueller, S.	[15]	+	+	−	+	+	+

Risk of bias legend: R: bias arising from the randomization process; D: bias due to deviations from intended interventions; Mi: bias due to missing outcome data; Me: bias in measurement of the outcome; S: bias in the selection of the reported result; O: overall risk of bias; +: Low; −: some concerns.

**Table 3:** Quality assessment in a systematic review of cohort studies, using the Newcastle-Ottawa Scale. References (Ref.) are available from the main document.

Study	Ref.	Selection	Comparability	Outcome
Crispo, A.L.	[9]	***	*	***
Lizotte, R.J.	[12]	***	**	**
VanderWeide, L.A.	[11]	**	*	**
Ludtke, K.A.	[16]	***	*	***
Wong, A.	[17]	***	**	***
Love, K.	[13]	***	**	***
Collier, T.E.	[14]	***	**	**

Risk of bias legend: a study can be awarded for a maximum cumulative number of nine stars (categories Selection max. 4 stars; categories Comparability max. 2 stars; categories Outcome max. 3 stars).

**Table 4:** PICOS method for selecting clinical studies in the systematic reviews.

Participants	Intervention	Comparison	Outcomes	Study Design
Adult patients in any setting with alcohol withdrawal syndrome	Dexmedetomidine as adjunctive therapy to standard of care	Standard of care	Primary outcomes: Tracheal intubation Secondary outcomes: (a) Hypotension (b) Bradycardia	Randomized controlled trials and observational studies (including cohort and case-control studies)

## Data Availability

The data that support the findings of this meta-analysis are available from the corresponding author (M.F.) upon reasonable request.

## References

[1] Association A.P. (2000). Diagnostic and Statistical Manual of Mental Disorders.

[2] Ganatra R.B., Breu A.C., Ronan M.V. (2022). Clinical guideline highlights for the hospitalist: 2020 American Society of Addiction Medicine clinical practice guideline on alcohol withdrawal management. J. Hosp. Med..

[3] Weerink M.A.S., Struys M.M.R.F., Hannivoort L.N., Barends C.R.M., Absalom A.R., Colin P. (2017). Clinical Pharmacokinetics and Pharmacodynamics of Dexmedetomidine. Clin. Pharmacokinet..

[4] Yuki K. (2021). The immunomodulatory mechanism of dexmedetomidine. Int. Immunopharmacol..

[5] U.S. Department of Health and Human Services (HHS), Substance Abuse and Mental Health Services Administration (SAMHSA), Center for Behavioral Health Statistics and Quality, Treatment Services Branch (2021). Preliminary Findings from Drug-Related Emergency Department Visits. https://store.samhsa.gov/sites/default/files/PEP22-07-03-001.pdf.

[6] Peterson C., Li M., Xu L., Mikosz C.A., Luo F. (2021). Assessment of Annual Cost of Substance Use Disorder in US Hospitals. JAMA Netw. Open.

[7] Chang D.W., Shapiro M.F. (2016). Association between Intensive Care Unit Utilization during Hospitalization and Costs, Use of Invasive Procedures, and Mortality. JAMA Intern. Med..

[8] Harrer M., Cuijpers P., Furukawa T.A., Ebert D.D. (2021). Doing Meta-Analysis with R: A Hands-On Guide.

[9] Crispo A.L., Daley M.J., Pepin J.L., Harford P.H., Brown C.V. (2014). Comparison of clinical outcomes in nonintubated patients with severe alcohol withdrawal syndrome treated with continuous-infusion sedatives: Dexmedetomidine versus benzodiazepines. Pharmacotherapy.

[10] Bielka K., Kuchyn I., Glumcher F. (2015). Addition of dexmedetomidine to benzodiazepines for patients with alcohol withdrawal syndrome in the intensive care unit: A randomized controlled study. Ann. Intensive Care.

[11] VanderWeide L.A., Foster C.J., MacLaren R., Kiser T.H., Fish D.N., Mueller S.W. (2016). Evaluation of Early Dexmedetomidine Addition to the Standard of Care for Severe Alcohol Withdrawal in the ICU:A Retrospective Controlled Cohort Study. J. Intensive Care Med..

[12] Lizotte R.J., Kappes J.A., Bartel B.J., Hayes K.M., Lesselyoung V.L. (2014). Evaluating the effects of dexmedetomidine compared to propofol as adjunctive therapy in patients with alcohol withdrawal. Clin. Pharmacol..

[13] Love K., Zimmermann A.E. (2020). Use of Propofol Plus Dexmedetomidine in Patients Experiencing Severe Alcohol Withdrawal in the Intensive Care Unit. J. Clin. Pharmacol..

[14] Collier T.E., Farrell L.B., Killian A.D., Kataria V.K. (2022). Effect of Adjunctive Dexmedetomidine in the Treatment of Alcohol Withdrawal Compared to Benzodiazepine Symptom-Triggered Therapy in Critically Ill Patients: The EvADE Study. J. Pharm. Pract..

[15] Mueller S.W., Preslaski C.R., Kiser T.H., Fish D.N., Lavelle J.C., Malkoski S.P., MacLaren R. (2014). A randomized, double-blind, placebo-controlled dose range study of dexmedetomidine as adjunctive therapy for alcohol withdrawal. Crit. Care Med..

[16] Ludtke K.A., Stanley K.S., Yount N.L., Gerkin R.D. (2015). Retrospective Review of Critically Ill Patients Experiencing Alcohol Withdrawal: Dexmedetomidine Versus Propofol and/or Lorazepam Continuous Infusions. Hosp. Pharm..

[17] Wong A., Benedict N.J., Kane-Gill S.L. (2015). Multicenter evaluation of pharmacologic management and outcomes associated with severe resistant alcohol withdrawal. J. Crit. Care.

[18] Riihioja P., Jaatinen P., Oksanen H., Haapalinna A., Heinonen E., Hervonen A. (1997). Dexmedetomidine, diazepam, and propranolol in the treatment of ethanol withdrawal symptoms in the rat. Alcohol. Clin. Exp. Res..

[19] Rovasalo A., Tohmo H., Aantaa R., Kettunen E., Palojoki R. (2006). Dexmedetomidine as an adjuvant in the treatment of alcohol withdrawal delirium: A case report. Gen. Hosp. Psychiatry.

[20] DeMuro J.P., Botros D.G., Wirkowski E., Hanna A.F. (2012). Use of dexmedetomidine for the treatment of alcohol withdrawal syndrome in critically ill patients: A retrospective case series. J. Anesth..

[21] Rayner S.G., Weinert C.R., Peng H., Jepsen S., Broccard A.F. (2012). Dexmedetomidine as adjunct treatment for severe alcohol withdrawal in the ICU. Ann. Intensive Care.

[22] Frazee E.N., Personett H.A., Leung J.G., Nelson S., Dierkhising R.A., Bauer P.R. (2014). Influence of dexmedetomidine therapy on the management of severe alcohol withdrawal syndrome in critically ill patients. J. Crit. Care.

[23] Polintan E.T.T., Danganan L.M.L., Cruz N.S., Macapagal S.C., Catahay J.A., Patarroyo-Aponte G., Azmaiparashvili Z., Lo K.B. (2022). Adjunctive Dexmedetomidine in Alcohol Withdrawal Syndrome: A Systematic Review and Meta-analysis of Retrospective Cohort Studies and Randomized Controlled Trials. Ann. Pharmacother..

[24] Fiore M., Torretta G., Passavanti M.B., Sansone P., Pace M.C., Alfieri A., Aurilio C., Simeon V., Chiodini P., Pota V. (2019). Dexmedetomidine as adjunctive therapy for the treatment of alcohol withdrawal syndrome: A systematic review protocol. JBI Database Syst. Rev. Implement. Rep..

[25] Page M.J., McKenzie J.E., Bossuyt P.M., Boutron I., Hoffmann T.C., Mulrow C.D., Shamseer L., Tetzlaff J.M., Akl E.A., Brennan S.E. (2021). The PRISMA 2020 statement: An updated guideline for reporting systematic reviews. BMJ.

[26] Sterne J.A.C., Savović J., Page M.J., Elbers R.G., Blencowe N.S., Boutron I., Cates C.J., Cheng H.-Y., Corbett M.S., Eldridge S.M. (2019). RoB 2: A revised tool for assessing risk of bias in randomised trials. BMJ.

[27] Wells G.A., Shea B., O’Connell D., Peterson J., Welch V., Losos M., Tugwell P. (2000). The Newcastle-Ottawa Scale (NOS) for Assessing the Quality of Nonrandomised Studies in Meta-Analyses.

[28] Mantel N., Haenszel W. (1959). Statistical aspects of the analysis of data from retrospective studies of disease. J. Natl. Cancer Inst..

[29] Hedges L.V., Vevea J.L. (1996). Estimating Effect Size under Publication Bias: Small Sample Properties and Robustness of a Random Effects Selection Model. J. Educ. Behav. Stat..

[30] Paule R.C., Mandel J. (1982). Consensus Values and Weighting Factors. J. Res. Natl. Bur. Stand..

